# Spatial patterns of continental shelf faunal community structure along the Western Antarctic Peninsula

**DOI:** 10.1371/journal.pone.0239895

**Published:** 2020-10-01

**Authors:** Alan M. Friedlander, Whitney Goodell, Pelayo Salinas-de-León, Enric Ballesteros, Eric Berkenpas, Andrea P. Capurro, César A. Cárdenas, Mathias Hüne, Cristian Lagger, Mauricio F. Landaeta, Alex Muñoz, Mercedes Santos, Alan Turchik, Rodolfo Werner, Enric Sala

**Affiliations:** 1 Pristine Seas, National Geographic Society, Washington, DC, United States of America; 2 Hawaiʿi Institute of Marine Biology, University of Hawaiʿi, Kāneʻohe, Hawaiʿi, United States of America; 3 Charles Darwin Research Station, Charles Darwin Foundation, Puerto Ayora, Galápagos, Ecuador; 4 Centre d'Estudis Avancats de Blanes-CSIC, Blanes, Girona, Spain; 5 Exploration Technology, National Geographic Society, Washington, DC, United States of America; 6 Instituto Antártico Argentino/Dirección Nacional del Antártico/Cancilleria Argentina, Buenos Aires, Argentina; 7 Departamento Científico, Instituto Antártico Chileno, Punta Arenas, Chile; 8 Fundación Ictiológica, Santiago, Chile; 9 Instituto de Diversidad y Ecología Animal (IDEA), CONICET-UNC and Facultad de Ciencias Exactas, Físicas y Naturales, Universidad Nacional de Córdoba, Córdoba, Argentina; 10 Laboratorio de Ictioplancton (LABITI), Escuela de Biología Marina, Facultad de Ciencias del Mar y de Recursos Naturales, Universidad de Valparaíso, Viña del Mar, Chile; 11 The Pew Charitable Trusts & Antarctic and Southern Ocean Coalition, Washington, DC, United States of America; Universidad de Antioquia, COLOMBIA

## Abstract

Knowledge of continental shelf faunal biodiversity of Antarctica is patchy and as such, the ecology of this unique ecosystem is not fully understood. To this end, we deployed baited cameras at 20 locations along ~ 500 km of the Western Antarctic Peninsula (WAP) at depths from 90 to 797 m. We identified 111 unique taxa, with mud bottom accounting for 90% of the dominant (≥ 50% cover) habitat sampled. Amphipoda comprised 41% of the total maximum number of individuals per camera deployment (MaxN) and occurred on 75% of deployments. Excluding this taxon, the highest MaxN occurred around King George/25 de Mayo Island and was driven primarily by the abundance of krill (Euphausiidae), which accounted for 36% of total average MaxN among deployments around this island. In comparison, krill comprised 22% of total average MaxN at Deception Island and only 10% along the peninsula. Taxa richness, diversity, and evenness all increased with depth and depth explained 18.2% of the variation in community structure among locations, which may be explained by decreasing ice scour with depth. We identified a number of Vulnerable Marine Ecosystem taxa, including habitat-forming species of cold-water corals and sponge fields. Channichthyidae was the most common fish family, occurring on 80% of all deployments. The Antarctic jonasfish (*Notolepis coatsorum*) was the most frequently encountered fish taxa, occurring on 70% of all deployments and comprising 25% of total MaxN among all deployments. Nototheniidae was the most numerically abundant fish family, accounting for 36% of total MaxN and was present on 70% of the deployments. The WAP is among the fastest warming regions on Earth and mitigating the impacts of warming, along with more direct impacts such as those from fishing, is critical in providing opportunities for species to adapt to environmental change and to preserve this unique ecosystem.

## Introduction

The Southern Ocean, surrounding Antarctica, is one of the least altered marine ecosystems on Earth. It encompasses 15% of the world’s oceans and is home to thousands of endemic species [1, 2]. Due to intense summer productivity, the region is responsible for ~20% of global atmospheric CO_2_ draw-down [3]. Despite its global importance, large areas of Antarctica have never been sampled and much of the biology and ecology is still poorly known [4–6], mainly due to difficulties associated with its remoteness and hostile weather and sea conditions, often making field operations problematic [7].

The Antarctic continental shelf covers more than 4.6 million km^2^ and compared with the rest of the world’s ocean shelfs, it is unusually deep (average ~ 450 m, max. > 1,000 m) because of scouring from ice shelves at previous glacial maxima and depression by the enormous mass of continental ice [8–10]. The average width of the shelf (~ 125 km) is almost twice that of shelves elsewhere in the world and constitutes about 11.4% of the world’s continental shelf area [9]. The shelf sediments are a combination of glacial deposits and diatomaceous muds [2], with one-third of the continental shelf covered by floating ice shelves [9].

The Western Antarctic Peninsula (WAP) is one of the most rapidly changing ecosystems on the planet and is an area of rich biodiversity, most of which has been described to lie on the continental shelf [4, 9]. The benthic fauna of the Antarctic continental shelf resides in a cold, well oxygenated, and oceanographically stable environment [4], at least since the last glacial maxima [11]. The unique geology, oceanography, and biogeography of the WAP continental shelf has resulted in a distinctive marine ecosystem, with some groups being over-represented (e.g., bryozoans, sponges, and amphipods), while others are under-represented (e.g., decapod crustaceans, bivalve molluscs, most groups of fishes) [12–14]. The modern benthic shelf fauna is characterized by the lack of active, skeleton-breaking (durophagous) predators such as crabs, lobsters, and many fishes, and the dominance in many areas of epifaunal suspension feeders [15, 16]. However, recent studies have reported the presence of the king crab (*Paralomis birsteini*) off the continental shelf of the WAP, with the potential to succesfully reproduce and which could radically alter the composition and trophic structure of the shelf-benthos in Antarctica [17, 18].

The fauna of the WAP continental shelf has been relatively well studied taxonomically [9, 13, 19, 20]; however, most studies have been conducted in areas close to research stations and mainly at depths shallower than 100 m, which are depths that are heavily affected by scouring produced by icebergs [5, 21, 22]. In addition, trawls, sledges, and dredges were historically the most common methods of sampling the shelf benthic marine communities of Antarctica [6, 23, 24]. While these methods are excellent for species identification of sessile and slow-moving benthic organisms, they are not as efficient at capturing more mobile species, are destructive, and cannot describe species behaviours and interactions present in the ecosystem. Advances in technology (e.g., photographic and video imagery, SCUBA, remotely operated vehicles, autonomous underwater vehicles) have increased the rate of new species discoveries for the WAP, as well as helping to develop a better understanding of ecosystem patterns and processes in the region [2, 25–29].

Previous studies in the region have described dense three-dimensional communities formed by sponges, hydrocorals, gorgonians, and ascidians that are important hotspots of biodiversity [30, 31]. Protecting these Vulnerable Marine Ecosystems (VMEs) is an important component of the framework for managing high seas bottom fisheries under the United Nations General Assembly Sustainable Fisheries Resolution [32]. In response to this, the Commission for the Conservation of Antarctic Marine Living Resources (CCAMLR) has also developed methods for identification and protection of VMEs through a range of conservation measures [33].

The Antarctic Peninsula ecosystem is changing rapidly due to the impact of climate change and increased temperatures, along with profound changes in the physical environment, including timing and reduction of sea ice, melting of ice shelves, retreat of coastal glaciers, and increased precipitation [34–37]. These dramatic changes are threatening these rich but fragile biological communities, where the roles and interactions amongst species is still poorly understood and thus leaving notable gaps in our understanding of climate change implications to Antarctic biodiversity, ecosystems, and their future conservation.

Since 2002, CCAMLR has been working on the development of a network of marine protected areas (MPAs) with the aim of conserving marine biodiversity in the Convention Area. Consistent with this goal and considering the uniqueness of the Western Antarctic Peninsula (WAP) and South Scotia Arc region, the delegations of Argentina and Chile proposed the establishment of an MPA in Planning Domain 1 (D1MPA), to protect representative habitats for marine living resources, preserve ecosystem processes, protect vital areas for keystone species, and designate areas for scientific research and monitoring (https://www.ccamlr.org/en/ccamlr-xxxvii/31). To support this effort, the governments of Chile and Argentina, in collaboration with National Geographic Pristine Seas, organized a bi-national expedition to the WAP in January 2019, with the aim of providing support to the MPA proposal put forward jointly by the two countries. To this end, we set out to explore the ecosystems of the continental shelf along the WAP and associated islands using National Geographic’s deep-sea cameras [38] to capture high quality imagery of areas of the Antarctic sea floor and the associated fauna, which have been comparatively less well explored. Our objective was to describe the distribution and abundance of benthic and demersal organisms along ~ 500 km of WAP coastline and characterize the spatial patterns in community structure, while also providing data to support the proposal by Argentina and Chile for an MPA for the WAP.

## Materials and methods

### Ethics statement

Data were collected by all authors in a collaborative effort. Non-invasive research was conducted, which included photographs as described in the methods below. The governments of Argentina and Chile granted all necessary permission to conduct this research. No vertebrate sampling was conducted and therefore no approval was required by any Institutional Animal Care and Use Committee. Our data are publicly available at OBIS/GBIF: https://obis.org/dataset/7afba27f-8547-4899-829e-b4bd25765322

### Location

**Fig 1:**
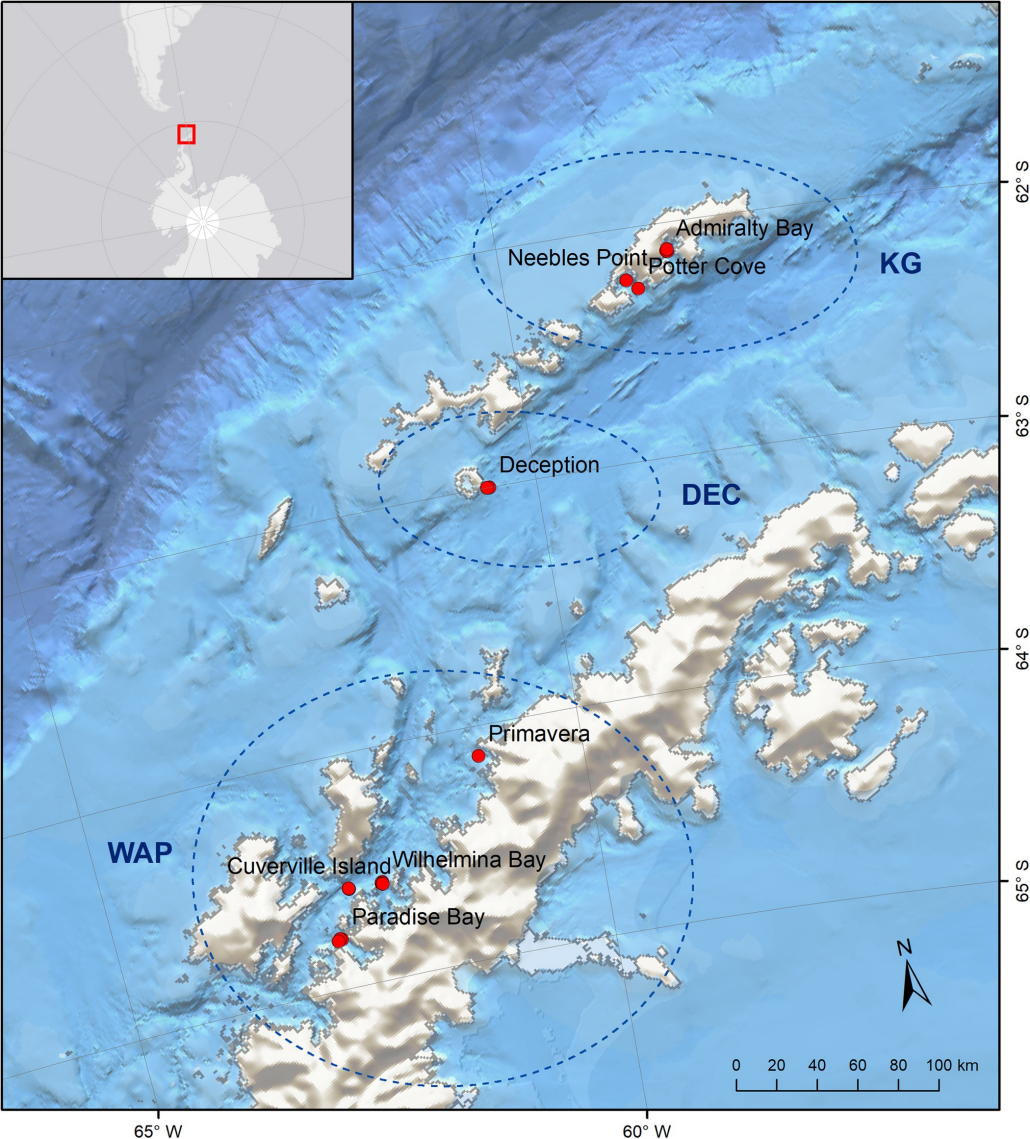
Sampling stations visited during the expedition. KG = King George/25 de Mayo Island (n = 5), DEC = Deception Island (n = 3), WAP = Western Antarctic Peninsula (n = 12).

The Antarctic Peninsula (AP) extends for ~1,300 km along the northernmost portion of the Antarctica continent and is ~ 1,000 km from the southern tip of South America, across the Drake Passage (Fig 1). The WAP, including the South Shetland Islands and the South Scotia Arc, is part of CCAMLR Planning Domain 1, a physical division in which the Convention Area is divided for management purposes (https://www.ccamlr.org/en/science/mpa-planning-domains).

### Deep-sea camera surveys

National Geographic’s deep-sea cameras were used to quantify marine life along the shelf of the WAP. These systems consist of high definition cameras (Sony Handycam FDR-AX33 4K Ultra-High Definition video with a 20.6 megapixel still image capability) in a 33-cm diameter borosilicate glass sphere that is rated to ~7,000 m depth [38]. Viewing area per frame for the cameras is ca. 17 m^2^, depending on the steepness of the slope where the camera lands. Cameras were baited with ~ 1 kg of frozen sardines and deployed for ~ three hrs. Lighting at depth was achieved through a high-intensity LED array. Depth gauging was accomplished using an internal logging pressure sensor. The cameras were weighted with a 12-kg locally procured biodegradable sandbag weight with a descent rate of ~1 m s^-1^. At the programmed time, sandbag weights were automatically released allowing the cameras to return to the surface. A total of 20 camera deployments were conducted in January 2019 in the study area, which were aggregated in three major areas: King George/25 de Mayo Island (KG, n = 5), Deception Island (DEC, n = 3), and along the Western Antarctic Peninsula (WAP, n = 12) (Fig 1).

Video footage was annotated for taxa present (identified to the lowest possible taxonomic level) and the maximum number of individuals of a given taxon per video frame (MaxN). Frequency of occurrence (Freq. occ. %) for each taxon observed was calculated as the percentage of incidence across all deployments. Taxa were classified as VME taxa based on CCAMLR [33]. The substrata for each camera deployment were classified into standard geological categories consisting of mud, pebble, cobble, and boulder [39]. Seafloor type was defined by the approximate percent cover of the two most prevalent substrata in each habitat patch. The first type was the substratum accounting for ≥ 50% of the patch, and the second most prevalent substratum accounted for an additional ≥ 30% of the patch.

### Statistical analyses

An index of relative dominance (IRD) for each taxon was created by multiplying the percent frequency of occurrence of a given taxon by the relative percent of MaxN of that taxon x 100 [40]. Due to the numerical dominance of Amphipoda in our samples, they were excluded from all analyses except for the comparisons of number of taxa. Total MaxN per deployment was calculated as the sum of MaxN for all taxa for that deployment. Species diversity was calculated from the Shannon-Weaver Diversity Index (Ludwig and Reynolds 1988): $H'=-\sum (p_{i}lnp_{i})$, where p_i_ is the proportion of all individuals counted that were of species i. Pielou’s evenness was calculated as: J = H´/ln(S), where S is the total number of species present. Comparison of community metrics (richness, MaxN, diversity, evenness) among locations were tested using a Kruskal-Wallis rank sum test with unplanned multiple comparisons performed using Dunn’s test for all pairs by joint ranking. Community metrics were related to deployment depth using least-squares linear regression.

Drivers of community structure were investigated using permutation-based multivariate analysis of variance (PERMANOVA). A Bray–Curtis similarity matrix was created from MaxN of each taxon for each deployment. Permutation of residuals was under a reduced model (Sums of squares Type III–partial) with 999 permutations used in the analysis. Location (KG, DEC, WAP) was treated as a fixed factor in the one-way PERMANOVA. Data were ln(x+1)-transformed prior to analysis. All species of krill (Euphausiidae) were pooled for analyses owing to the difficulty in distinguishing them in video annotation.

Principal Coordinate Analysis (PCO) was used to display community structure among locations in ordination space. The primary taxa vectors driving the ordination (Pearson product-moment correlations *r* ≥ 0.5) were overlaid on the PCO plot to visualize the major taxa that explained the spatial distribution patterns observed. Interpretation of PERMANOVA results was aided using individual analysis of similarities (ANOSIM), distance-based linear modelling (DistLM), and similarity percentages analysis (SIMPER) of species responsible for such patterns [41]. SIMPER identified the taxa most responsible for the percentage dissimilarities between locations using Bray-Curtis similarity analysis of hierarchical agglomerative group average clustering [41]. All PERMANOVA, PCO, and SIMPER analyses were conducted using Primer v6.

## Results

Deep drop-camera deployments ranged in depth from 90 to 797 m ($\bar{X}$ = 421.9±227.3 sd) (Tables 1 and S1). Mud bottom accounted for 90% of the dominant (≥ 50% cover) habitat type, with cobble and pebble each present at only one site. Mud also accounted for 70% of the secondary habitat type (≥ 30% and < 50% cover), with pebble and boulder habitat each present at three sites.

**Table 1:** Metadata for deep-sea camera deployments.

Location	N	Average depth (m)	Max depth (m)
Western Antarctic Peninsula	12	503.2 (228.8)	797
King George/25 de Mayo*	5	235.0 (146.3)	456
Deception Island	3	408.3 (180.9)	599
Total	20	421.9 (227.3)	797

One standard deviation of the mean in parentheses.

*Argentine recognized name. N = number of deployments.

### Community characteristics

**Fig 2:**
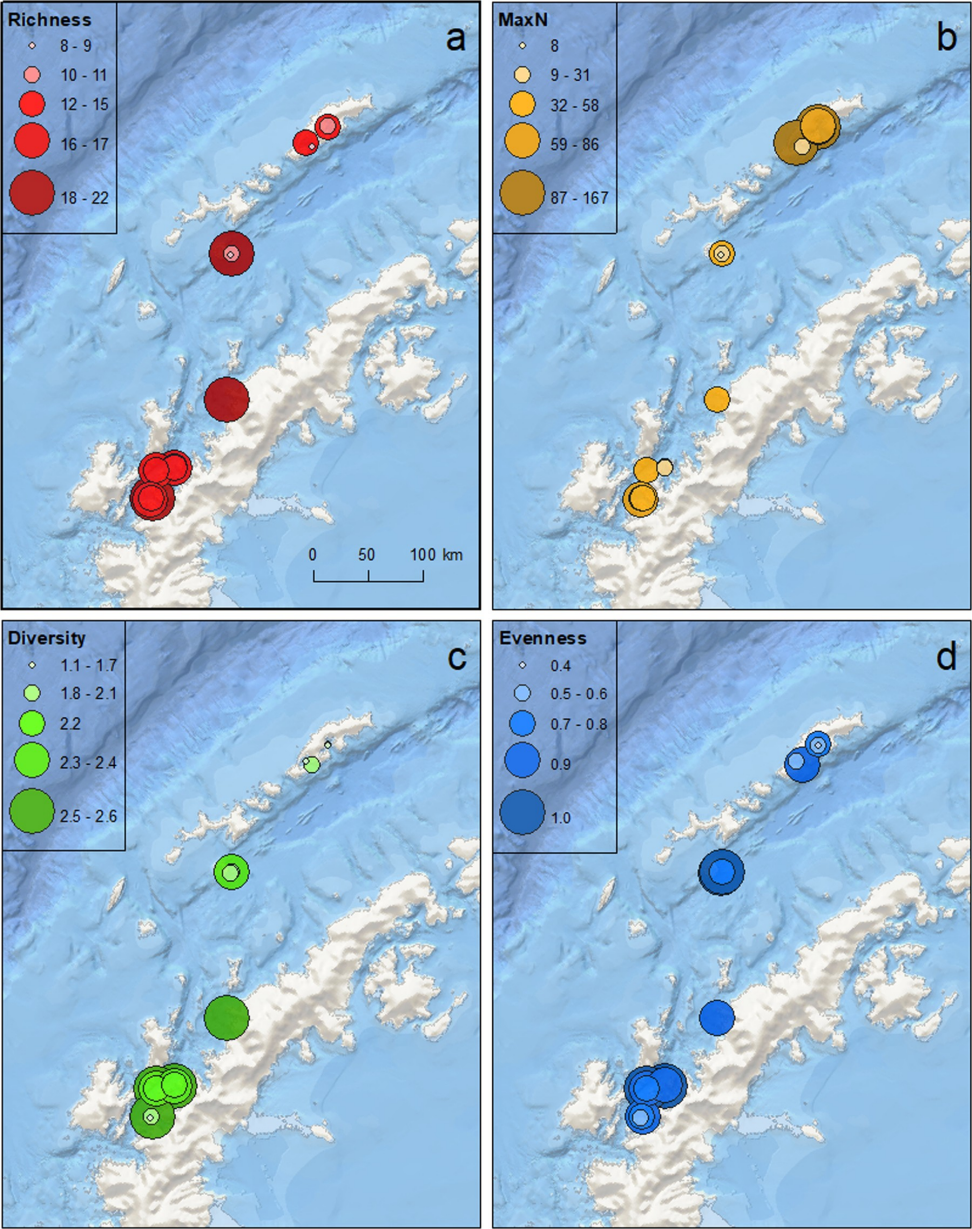
Community metrics from dee-sea camera deployments along the Western Antarctic Peninsula and associated islands. A. Species richness, B. MaxN–sum of the maximum number of individuals per deployment, excluding Amphipoda, C. Shannon-Weaver Diversity, and D. Pielou’s Evenness also calculated excluding Amphipoda.

We identified 111 unique taxa on our camera deployments, representing 11 phyla, 24 classes, 40 orders, and 42 families (S2 Table). Invertebrates accounted for 76 unique taxa, with fishes accounting for 33 taxa. Additionally, one Gentoo penguin (*Pygoscelis papua*) and one leopard seal (*Hydrurga leptonyx*) were observed on the cameras, at 90 and 178 m depths, respectively. The mean number of taxa per deployment was 14.50 (± 3.49 sd), with a minimum of 9 and a maximum of 20 taxa observed among all deployments (Table 2). The number of taxa per deployment was not significantly different among the three sampling locations (KG, DEC, and WAP, χ^2^ = 4.60, p = 0.100), although richness tended to be lower at KG and increased along the WAP (Fig 2A).

**Table 2:** Community metrics from deep-sea camera deployments.

Metric	KG	DEC	WAP	χ^2^	p	Multiple comps.
Richness	12.40 (2.41)	12.33 (5.90)	15.92 (2.68)	4.60	0.100	
MaxN	114.40 (28.34)	52.33 (36.59)	88.00 (18.29)	2.73	0.256	
MaxN w/o	96.40 (59.08)	21.67 (15.82)	42.67 (18.17)	7.78	0.020	KG WAP DEC
Amphipoda						^______________^
Diversity	1.55 (0.29)	2.11 (0.23)	2.27 (0.32)	9.01	0.011	WAP DEC KG
						^____________^
Evenness	0.65 (0.17)	0.91 (0.08)	0.84 (0.11)	6.43	0.040	DEC WAP KG
						^____________^

MaxN—maximum number of individuals of a given taxon per video frame. Shannon-Weaver Diversity and Pielou’s Evenness were calculated without Amphipoda. One standard deviation of the mean in parentheses. χ^2^ –Chi Square approximation for Kruskal-Wallis rank sum test. Dunn’s Multiple Comparisons tests were used for unplanned multiple comparisons among locations. Underlined locations are not significantly different at α = 0.05.

Amphipoda accounted for 41% of the total MaxN and occurred on 75% of the deployments. Total average MaxN for all taxa, including Amphipoda, was 89.25 (± 63.05 sd) and did not differ significantly among locations (Table 2). However, when Amphipoda were excluded, total taxa average MaxN was 52.95 (± 40.87) and differed significantly among locations (χ^2^ = 7.78, p = 0.02), with the highest total average MaxN at KG and the lowest at DEC (Fig 2B). Diversity without Amphipoda was also significantly different among locations, with the highest diversity along the WAP and the lowest at KG (Fig 2C). Evenness without Amphipoda was also significantly different among locations, with the highest evenness at DEC and the lowest at KG (Fig 2D). Diversity increased significantly with deployment depth (p = 0.039, Table 3). Taxa richness and evenness also increased slightly with depth, but these trends were not significant (p = 0.33 and p = 0.18, respectively). Total MaxN across all taxa and total MaxN excluding Amphipoda declined with depth but not significantly (p = 0.15 and p = 0.27, respectively). Average deployment depth was highest at WAP ($\bar{X}$ = 503.17 ± 228.81), followed by DEC ($\bar{X}$ = 408.33 ± 180.95) and KG ($\bar{X}$ = 235.00 ± 146.20). However, these differences were not significant (χ^2^ = 5.53, p = 0.063).

**Table 3:** Relationships between community metrics and deployment depth using least-squares linear regression analyses.

Metric	Intercept	Slope	R^2^	F	p
Richness	12.989	0.003	0.055	1.037	0.332
Total MaxN	128.843	-0.094	0.114	2.326	0.145
Total MaxN w/o	72.693	-0.047	0.068	1.307	0.268
Amphipoda					
Diversity	1.700	0.001	0.215	4.941	0.039
Evenness	0.716	0.0002	0.097	1.937	0.181

Diversity and Evenness were calculated without Amphipoda.

**Fig 3:**
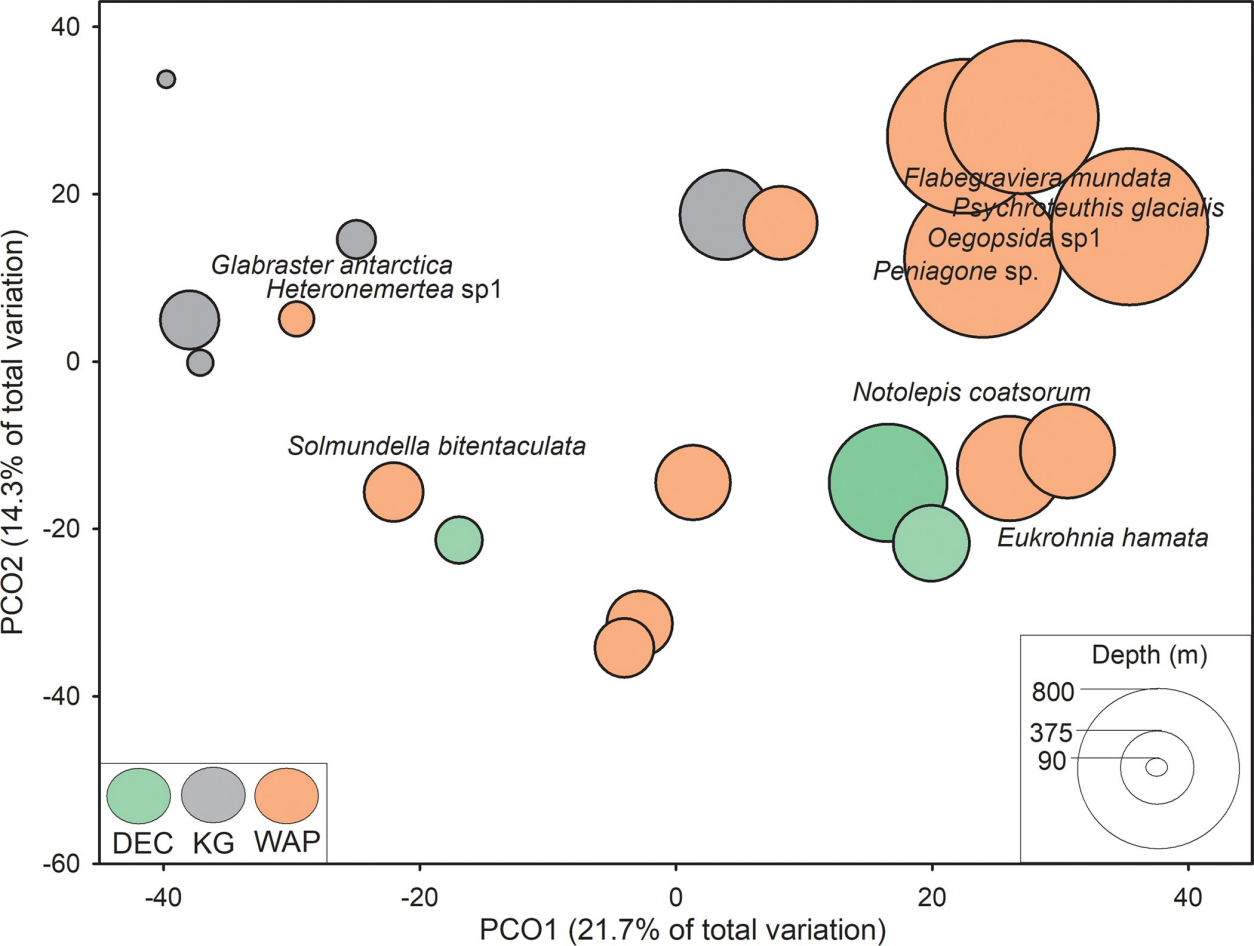
Principal coordinates analysis of community composition based on MaxN by deployment. Data were ln(x+1)-transformed prior to analyses. Vectors are the relative contribution and direction of influence of taxa to the observed variation among sites (Pearson product-moment correlations ≥ 0.5).

Deployment locations clustered in ordination space by location and depth (Fig 3). PCO1 accounted for 21.7% of total variation in faunal community composition, while PCO2 explained an additional 14.3% of the variation. Deeper locations, primarily along the WAP, clustered towards the upper right-hand side of the plot, while shallower locations, primarily at KG, clustered towards the lower end of PCO1. Four taxa were strongly correlated with deeper locations, primarily along the WAP. These included: glacial squid *Psychroteuthis glacialis*, the polychaete worm *Flabegraviera mundata*, the deep-sea benthic sea cucumber *Peniagone* sp., and the pelagic squid *Oegopsida* sp1. Taxa most closely correlated with shallow locations, primarily at KG were the Antarctic sea star *Glabraster antarctica*, and the nemertean worm *Heteronemertea* sp1.

There was a significant difference in community structure among locations, with KG significantly different from the other two locations, which were not different from one another (Pseudo-F_2,19_ = 1.983, p = 0.005, Table 4). Depth explained 18.2% of the variation in community structure (DistLM Pseudo-F_1,18_ = 4.00, p = 0.001).

**Table 4:** Comparison of community composition based on MaxN among locations using a Permutation-Based Multivariate Analysis of Variance (PERMANOVA).

Source	df	SS	MS	Pseudo-F	P(perm)
Location	2	10586	5292.8	1.931	0.005
Residual	17	45372	2669.0		
Total	19	55958			
Groups	Statistic	p			
King George, Deception	0.590	0.018			
King George, Peninsula	0.381	0.007			
Deception, Peninsula	-0.022	0.451			

Date were ln(x+1) transformed prior to analysis.

### Taxa characteristics

**Fig 4:**
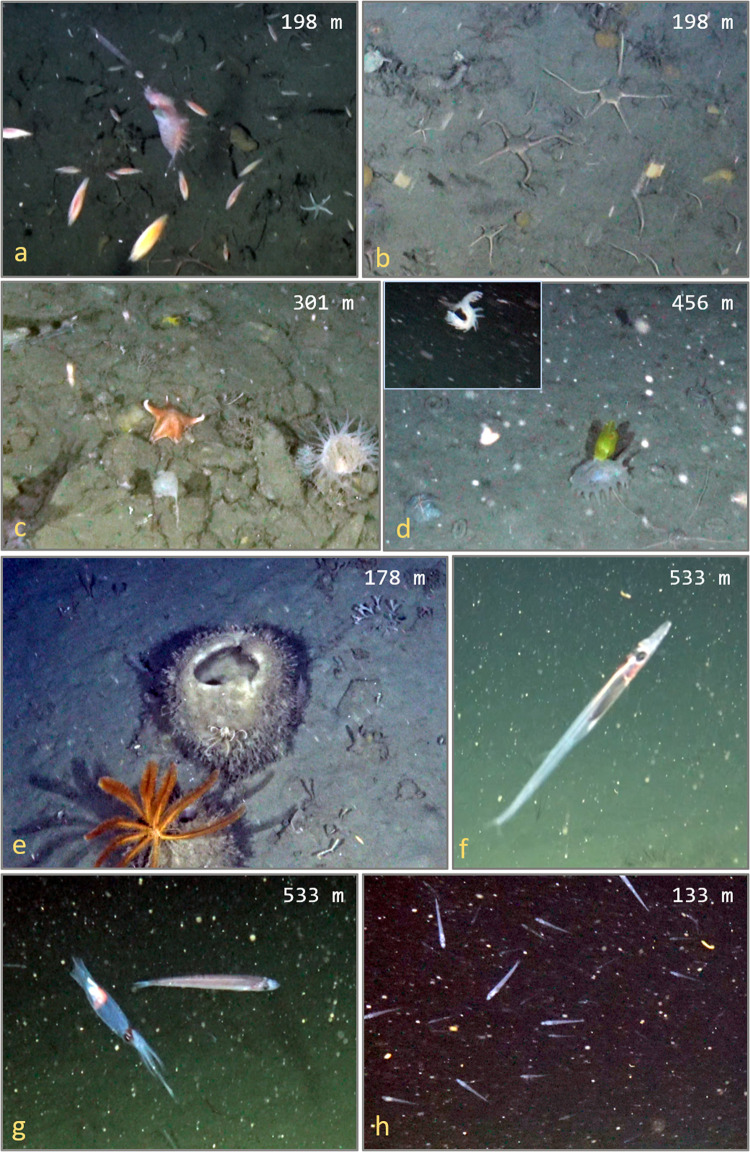
Common and important taxa observed on dropcam deployments. a. Euphausiidae (likely *Euphausia superba*) and Amphipoda, b. *Ophionotus victoriae*, c. (center) *Glabraster antarctica*, d. *Peniagone* sp.(inset: active swimming), e. *Rossella* sp. and *Solanometra antarctica*, f. *Notolepis coatsorum*, g. (left) *Psychroteuthis glacialis*, (right) *Pleuragramma antarctica*, h. Numerous *Pleuragramma antarctica*.

The taxa Amphipoda accounted for ~41% of all observed individuals and occurred in 75% of the deployments (Tables 5 and S1). Krill (Euphausiidae) were observed on 19 of the 20 deployments, accounting for 13.0% of average MaxN, with a maximum MaxN of 120 individuals per frame (Fig 4). Most of these krill individuals were identified as *Euphausia superba*, but other species such as *Euphausia crystallorophias* were observed but difficult to differentiate in counts. The brittle star *Ophionotus victoriae* comprised 9.8% of average MaxN and was present on 55% of the deployments. The Antarctic jonasfish (*Notolepis coatsorum*) was present of 70% of the deployments and comprised an additional 2.9% of average MaxN.

**Table 5:** Top 15 taxa overall among all 20 deployment locations.

Order	Family	Genus species	MaxN (sd)	Max. MaxN	% MaxN	% Freq	IRD
Amphipoda		Amphipoda	36.30 (54.15)	200	40.67	75	3050.4
Ophiurida	Ophiuridae	*Ophionotus victoriae*	8.75 (17.43)	60	9.80	55	539.2
Euphausiacea	Euphausiidae	Euphausiidae sp3	6.75 (26.14)	120	7.56	30	226.9
Aulopiformes	Paralepididae	*Notolepis coatsorum*	2.6 (3.17)	13	2.91	70	203.9
Euphausiacea	Euphausiidae	Euphausiidae sp1	2.85 (8.75)	40	3.19	25	79.8
Perciformes	Nototheniidae	*Pleuragramma antarctica*	2.15 (6.66)	30	2.41	30	72.3
Stolidobranchia	Pyuridae	*Pyura bouvetensis*	1.50 (2.57)	10	1.68	40	67.2
Euphausiacea	Euphausiidae	Euphausiidae sp2	1.60 (3.15)	11	1.79	35	62.7
Phragmophora	Eukrohniidae	*Eukrohnia hamate*	0.80 (0.68)	2	0.90	65	58.3
Terebellida	Flabelligeridae	*Flabegraviera mundata*	2.35 (5.32)	20	2.63	20	52.7
Oegopsida	Psychroteuthidae	*Psychroteuthis glacialis*	0.95 (1.33)	4	1.06	40	42.6
Perciformes	Nototheniidae	Nototheniidae	0.80 (1.17)	4	0.90	40	35.9
Elasipodida	Elpidiidae	*Peniagone* sp.	1.10 (2.31)	8	1.23	25	30.8
Perciformes	Channichthyidae	Channichthyidae sp1	0.65 (1.02)	3	0.73	35	25.5
Cidaroida	Ctenocidaridae	*Ctenocidaris perrieri*	0.6 (1.32)	6	0.67	35	23.5

MaxN = average maximum number of individuals per frame for that taxa among all deployments. Max. MaxN = maximum MaxN for that taxa. % Freq. = percent frequency of occurrence (n = 20). Taxa are ordered by index of relative dominance (IRD) = (% Freq. x % MaxN).

Average dissimilarity between locations based on SIMPER analyses was highest between KG and DEC (90.1%) and lowest between DEC and WAP (78.4%, Table 6). Abundance of Euphausiidae based on MaxN was an order of magnitude greater at KG compared to DEC and WAP. Abundance of the brittle star *Ophionotus victoriae* was 3.2 times higher at KG compared with WAP and 60.0 times higher than at DEC. MaxN of the Antarctic jonasfish (*Notolepis coatsorum*) was two times higher at DEC compared with WAP and accounted for the greatest dissimilarity (9.7%) between these two locations. This species was 14 times more abundant at DEC, based on MaxN, compared with KG and accounted for 5.7% of the dissimilarity between these two locations.

**Table 6:** Similarity of Percentages (SIMPER) for taxa most responsible for the percent dissimilarities between locations using Bray-Curtis similarity analysis of hierarchical agglomerative group average clustering.

A. Avg. dissimilarity = 90.07	KG	DEC	Diss.	% contrib.
Euphausiidae	34.80 (50.15)	4.67 (3.79)	19.78 (0.87)	21.96
*Ophionotus victoriae*	19.80 (23.10)	0.33 (0.58)	15.92 (1.25)	17.68
Alcyonacea sp2	8.00 (17.89)	0	8.6 (0.48)	9.55
*Pleuragramma antarctica*	7.60 (12.99)	0	7.92 (0.55)	8.80
*Notolepis coatsorum*	0.40 (0.89)	5.67 (6.58)	5.16 (1.03)	5.73
B. Avg. dissimilarity = 86.93	KG	WAP	Diss.	% contrib.
Euphausiidae	34.80 (50.15)	4.33 (2.77)	17.30 (0.85)	19.91
*Ophionotus victoriae*	19.80 (23.10)	6.25 (16.55)	13.87 (1.18)	15.95
Alcyonacea sp2	8.00 (17.89)	0	7.06 (0.49)	8.12
*Pleuragramma antarctica*	7.60 (12.99)	0.42 (0.67)	6.59 (0.59)	7.58
*Flabegraviera mundata*	0	3.92 (6.63)	3.21 (0.57)	3.69
C. Avg. dissimilarity = 78.43	DEC	WAP	Diss.	% contrib.
*Notolepis coatsorum*	5.67 (6.58)	2.75 (2.26)	7.61 (1.39)	9.71
*Ophionotus victoriae*	0.33 (0.58)	6.25 (16.55)	6.94 (0.45)	8.84
*Flabegraviera mundata*	0	3.92 (6.63)	6.05 (0.61)	7.71
Euphausiidae	4.67 (3.79)	4.33 (2.77)	5.60 (0.89)	7.13
*Tomopteris* sp.	2.00 (1.73)	0.33 (0.65)	2.96 (1.30)	3.78

SIMPER results exclude Amphipoda. Values are average MaxN with standard deviations in parentheses. Diss. = Average dissimilarity with one standard deviation of the mean in parentheses. Pair-wise comparisons: A = KG and DEC, B = KG and WAP, and C = DEC and WAP.

Crocodile icefishes (Channichthyidae) was the most frequently occurring family of fishes, being observed on 80% of all deployments. The Antarctic jonasfish (*Notolepis coatsorum*) was the most frequently occurring individual taxon of fish, occurring on 70% of the camera deployments and comprising 25% of total average MaxN among all deployments. Ice codfish (Nototheniidae) was the most abundant fish family, accounting for 36% of total average MaxN among all deployments and present on 70% of the deployments.

**Fig 5:**
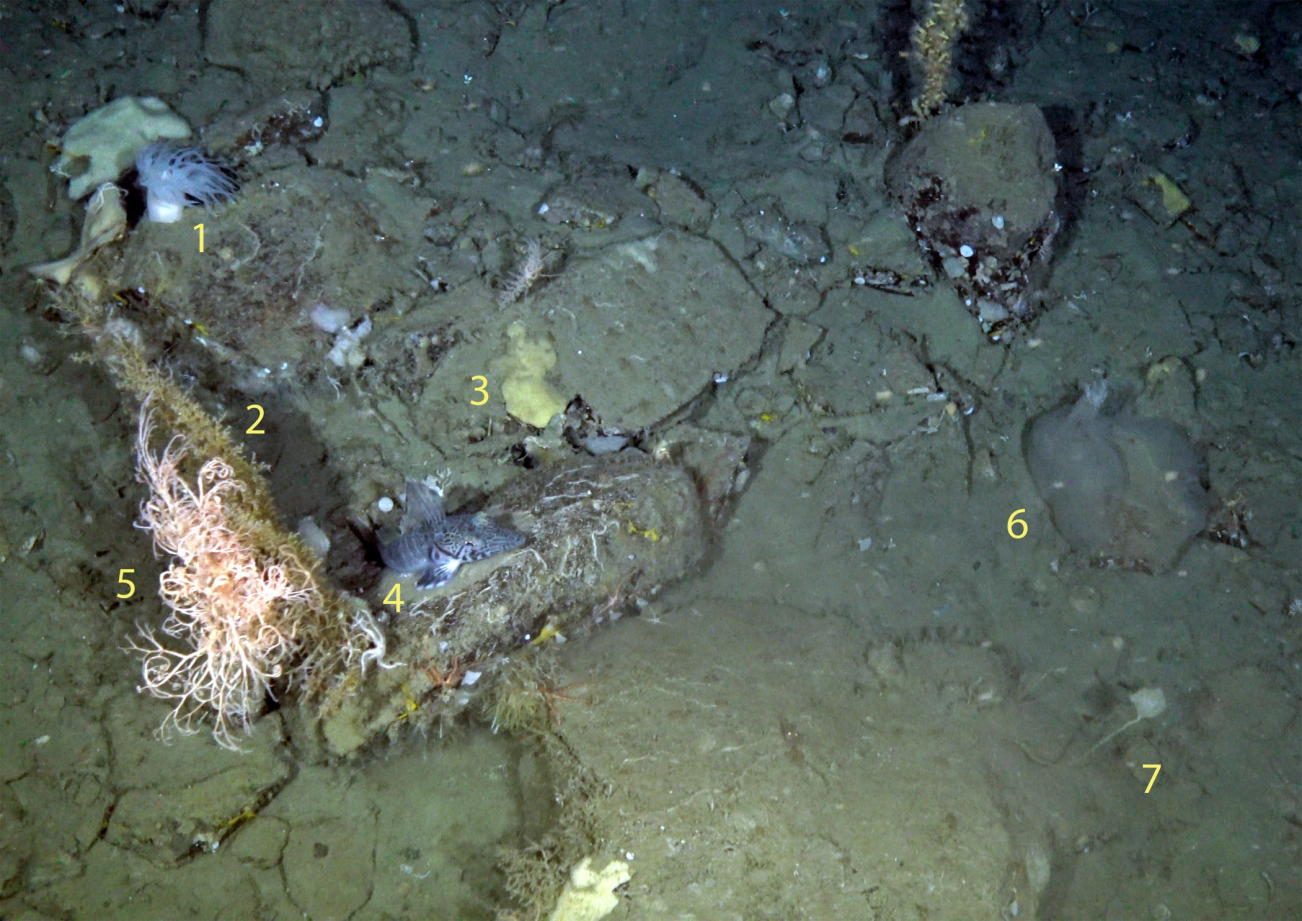
Deep-sea camera image from Wilhelmina Bay, WAP at 301 m. VME—Vulnerable Marine Ecosystem taxa. 1. *Actiniaria* sp. (VME), 2. Primnoidea (VME), 3. Demospongiae (VME), 4. *Pagetopsis macropterus*, 5. *Gorgonocephalus chilensis* (VME), 6. Holothuriidae, 7. *Pyura bouvetensis* (VME).

We identified a number of taxa that are classified as VME taxa (Fig 5). These include cold-water corals and sponge fields, which provide important habitat for a diversity of marine organisms. Sea urchins (Order: Cidaroida, likely *Ctenocidaris perrieri*), sea fans (Family: Primnoidae), and large glass sponges (*Rossella* spp.) were some of the most common VME taxa observed.

## Discussion

We used baited cameras to examine benthic and demersal communities along ~500 km of the WAP and associated islands from depths of 90 to 797 m. Our camera deployments allowed us to identify more than 100 taxa of benthic and demersal organisms and quantify their relative abundances. This non-invasive sampling tool can record information for long time intervals, providing important information on the abundance, community structure, and behaviour of sessile and mobile organisms, which is limited, as most studies in the region have used conventional sampling such as trawls and dredges that does not provide for *in situ* observations. Surprisingly, taxa richness recorded on our cameras is comparable to values obtained using the same camera system and methodology in the Tropical Eastern Pacific [42].

Our results show distinct differences in community structure among locations, with King George/25 de Mayo Island (KG) significantly different from Deception Island and the WAP. KG had the lowest taxa richness, diversity, and evenness but the highest MaxN, which was driven primarily by the abundance of krill. Taxa richness and diversity were highest along the WAP and diversity was significantly higher at deeper locations, which were primarily along the WAP. These patterns are somewhat confounded by the fact that the shallowest deployments were located at KG and the deepest deployments were along the WAP, although these differences were not significant. The influence of depth on the community is most probably explained by a decrease of ice scour with increased depth, which is the main physical disturbance affecting Antarctic benthic communities [10, 21, 22]. Previous studies have reported distinct patterns between northern areas of the Scotia Sea and the WAP associated with seabed disturbance produced by icebergs, but also due to differences in seabed temperature produced by the complex interactions between the cold waters of the Weddell Gyre and the warm waters of the Antarctic Circumpolar Current [43].

It is widely recognised that bottom fishing gear can cause extensive damage to the benthos, especially benthic invertebrates that form fragile biogenic structures, and numerous policies have been enacted to help protect these VMEs [32, 44]. Sea urchins, gorgonians, corals, and sponges were among the most common VME taxa we observed. CCAMLR has defined a VME to include the presence of benthic invertebrates that significantly contribute to the creation of complex three-dimensional structure, cluster in high densities, change the structure of the substratum, provide substrata for other organisms, or are rare or unique [33]. CCAMLR has adopted conservation measures aimed at minimizing adverse impacts on VMEs by fishing gear and other activities [32]. CCAMLR Conservation Measure 22–07 requires fishing vessels to monitor by-catch for the presence of VME taxa and report this information to the Commission. Quantifying the occurrence and abundance of VME indicator taxa provides a baseline from which these efforts can be evaluated and is critical in ensuring that these conservation measures are effective. Cameras deployed in the South Orkney Islands Southern Shelf MPA found the benthic assemblages of the area to be strongly correlated with seafloor texture, where hard bottom hosted a greater number of individuals, taxa and biomass with a dominance of filter feeding VME taxa [45]. Fishes were a relatively common component of the observed demersal community, but richness was low overall. The Antarctic ichthyofauna is limited and less diverse than might be expected, given the size and age of the Antarctic marine ecosystem [46, 47], with notothenoids accounting for the majority of the ichthyofauna in terms of species and biomass [48]. Ice codfish (Nototheniidae) and crocodile icefishes (Channichthyidae) were the most abundant fish families observed on camera deployments. Isolation and freezing water temperatures in the Southern Ocean limit the diversity of fish species in Antarctica and results in a very distinct ichthyofauna with unique adaptations. These adaptations include antifreeze glycoproteins in ice codfish that prevent their blood from freezing, the absence of haemoglobin in crocodile icefishes, and the lack of a heat shock response in certain species [47, 49]. These adaptations allow these families to survive in the absence of competitors. Despite low species richness, the region is a present-day hotspot of fish species formation and is dominated by the radiation of highly specialized and geographically restricted species (e.g. Nototheniidae). These hotspots have the fastest rates of speciation for marine fishes of any region on Earth [50], and it is unclear how climate change will affect these novel evolutionary processes.

Climate change is a major threat to the long-term survival of Antarctic marine communities [4]. Since Antarctic organisms have evolved in a very cold and stable environment, most species are expected to show limited capacity to tolerate even slight increases in seawater temperature [49, 51]. The rapid warming of high-latitude ecosystems can have major implications for fisheries, including the Antarctic krill fishery in the Southern Ocean. A recent study has shown that the distribution of krill has contracted southward during the past 90 years [52]. This changing distribution is already altering Antarctic food webs that rely heavily on krill and could have an impact on biogeochemical cycling. Projected seafloor warming is expected to produce a reduction in suitable habitats and significantly shift species distribution depending on whether they respond positively or negatively to warming [53]. Recent experimental research has demonstrated that warming by 1°C can have significant effects at the community level, reducing species diversity and species interactions [54].

The rapid regional warming along the WAP has led to profound changes in the cryosphere, which is causing environmental shifts that may severely affect pelagic and benthic communities in the region [55–58]. Discharge of sediment-laden melt water associated with massive ice loss can have negative consequences to the entire food web [59]. Conversely, material re-suspended by ploughing icebergs serves as an additional food source for benthic filter feeders that are characteristic of modern Antarctic benthic communities [11]. There are many unknowns as to how communities will respond to climate change, and studies like ours could help to better understand the current spatial variability in Antarctic shelf fauna and serve as a baseline for future comparisons.

### Conservation

Recognizing the value of MPAs in supporting ecosystem health by reducing overfishing and impacts to benthic habitats, CCAMLR became the first international body to commit to creating an MPA network. Although the Antarctic Treaty Consultative Meeting has established several small Antarctic Specially Protected Areas (ASPAs), they are mainly terrestrial, and only a few include small marine components. Because of the small area protected, the current ASPAs are considered inadequate to protect the Peninsula’s krill populations, millions of breeding seabirds, marine mammals, and the greater ecosystem [60].

CCAMLR has adopted a framework for the establishment of MPAs, which relies on the best available science and aims to conserve biodiversity, protect key ecosystem processes, establish scientific reference areas, among other conservation objectives [61]. In 2018, Chile and Argentina presented a formal joint proposal for the creation of D1MPA to CCAMLR [62]. The D1MPA would protect biodiversity hotspots, representative and unique benthic and pelagic habitats, as well as habitats and nursery areas for commercially and ecologically important fish species (e.g., icefish, silverfish, and toothfish), which have been exploited in the past [62]. The designation of the D1MPA would be key to meeting spatial conservation objectives of the Convention, contributing to the representative system of MPAs within CCAMLR.

Our study has identified VMEs, established baseline abundance estimates for important species, and has helped to better describe community structure along the WAP. As a result, our findings provide a valuable contribution in helping to inform MPA zoning for the discussions of the D1MPA. The conservation of this region, one of the most impacted and fastest changing regions of the Antarctic, remains one of the ultimate challenges for CCAMLR and studies like ours contributes to this conservation effort.

## Supporting information

S1 Table Deep-sea camera deployment information. (DOCX)

S2 Table Taxa observed on deep-sea camera deployments along the Antarctic Peninsula. (DOCX)
