# Supplementary material for: Spatial patterns of continental shelf faunal community structure along the Western Antarctic Peninsula
Source: PLoS One. 2020 Oct 1;15(10):e0239895. doi: 10.1371/journal.pone.0239895 (PMC7529263; doi:10.1371/journal.pone.0239895)
Supplement: S1 Table — (DOCX) [file pone.0239895.s001.docx]

S1 Table. Deep-sea camera deployment information. Lat. = latitude, Long. = longitude. Coordinate system = WGS 1984. Benthic cover: B = boulder, C = cobble, M = mud, P = pebble.

| Loc. | Lat. | Long. | Site | Date | Depth (m) | Benthic 50% | Benthic 30% |
| --- | --- | --- | --- | --- | --- | --- | --- |
| 1 | -62.221 | -58.866 | Neebles Point | 09-Jan-19 | 198 | M | M |
| 2 | -62.264 | -58.768 | Potter Cove | 10-Jan-19 | 456 | M | M |
| 3 | -63.000 | -60.448 | Deception | 13-Jan-19 | 599 | M | P |
| 4 | -63.000 | -60.448 | Deception | 13-Jan-19 | 387 | M | P |
| 5 | -63.000 | -60.468 | Deception | 13-Jan-19 | 239 | P | P |
| 6 | -64.784 | -62.749 | Paradise Bay | 15-Jan-19 | 533 | M | M |
| 7 | -64.780 | -62.769 | Paradise Bay | 15-Jan-19 | 479 | M | M |
| 8 | -64.790 | -62.789 | Paradise Bay | 15-Jan-19 | 374 | M | M |
| 9 | -64.129 | -61.033 | Primavera | 16-Jan-19 | 178 | M | B |
| 10 | -64.579 | -62.227 | Wilhelmina Bay | 17-Jan-19 | 301 | M | B |
| 11 | -64.582 | -62.236 | Wilhelmina Bay | 17-Jan-19 | 335 | M | B |
| 12 | -64.585 | -62.231 | Wilhelmina Bay | 17-Jan-19 | 301 | C | M |
| 13 | -64.587 | -62.229 | Wilhelmina Bay | 17-Jan-19 | 381 | M | M |
| 14 | -64.577 | -62.571 | Cuverville Island | 18-Jan-19 | 797 | M | M |
| 15 | -64.577 | -62.571 | Cuverville Island | 18-Jan-19 | 796 | M | M |
| 16 | -64.574 | -62.573 | Cuverville Island | 18-Jan-19 | 784 | M | M |
| 17 | -64.574 | -62.573 | Cuverville Island | 18-Jan-19 | 779 | M | M |
| 18 | -62.125 | -58.454 | Admiralty Bay | 20-Jan-19 | 298 | M | M |
| 19 | -62.121 | -58.465 | Admiralty Bay | 20-Jan-19 | 90 | M | M |
| 20 | -62.114 | -58.456 | Admiralty Bay | 20-Jan-19 | 133 | M | M |
