# Supplementary material for: Spatial patterns of continental shelf faunal community structure along the Western Antarctic Peninsula
Source: PLoS One. 2020 Oct 1;15(10):e0239895. doi: 10.1371/journal.pone.0239895 (PMC7529263; doi:10.1371/journal.pone.0239895)
Supplement: S2 Table — (DOCX) [file pone.0239895.s002.docx]

S2 Table. Taxa observed on deep-sea camera deployments along the Western Antarctic Peninsula. Freq. = frequency of occurrence (n=20).

| Phylum | Class | Order | Family | Genus species | Freq |
| --- | --- | --- | --- | --- | --- |
| Annelida | Polychaeta | Phyllodocida | Tomopteridae | *Tomopteris* sp. | 5 |
| Annelida | Polychaeta | Terebellida | Flabelligeridae | *Flabegraviera mundata* | 4 |
| Arthropoda | Malacostraca | Amphipoda |  | Amphipoda | 15 |
| Arthropoda | Malacostraca | Euphausiacea | Euphausiidae | *Euphausia crystallorophias* | 4 |
| Arthropoda | Malacostraca | Euphausiacea | Euphausiidae | Euphausiidae | 7 |
| Arthropoda | Malacostraca | Euphausiacea | Euphausiidae | Euphausiidae sp1 | 5 |
| Arthropoda | Malacostraca | Euphausiacea | Euphausiidae | Euphausiidae sp2 | 4 |
| Arthropoda | Malacostraca | Euphausiacea | Euphausiidae | Euphausiidae sp3 | 6 |
| Bryozoa | Gymnolaemata | Cheilostomatida |  | Bryozoan sp1 | 1 |
| Chaetognatha | Sagittoidea | Phragmophora | Eukrohniidae | *Eukrohnia hamata* | 13 |
| Chordata | Actinopterygii |  |  | Unknown fish | 6 |
| Chordata | Actinopterygii |  |  | Unknown fish sp3 | 1 |
| Chordata | Actinopterygii |  |  | Unknown fish sp4 | 1 |
| Chordata | Actinopterygii |  |  | Unknown fish sp5 | 1 |
| Chordata | Actinopterygii | Aulopiformes | Paralepididae | *Notolepis coatsorum* | 14 |
| Chordata | Actinopterygii | Myctophiformes | Myctophidae | *Gymnoscopelus* sp. | 2 |
| Chordata | Actinopterygii | Myctophiformes | Myctophidae | Myctophidae sp1 | 2 |
| Chordata | Actinopterygii | Myctophiformes | Myctophidae | Myctophidae sp2 | 3 |
| Chordata | Actinopterygii | Myctophiformes | Myctophidae | Myctophidae sp3 | 2 |
| Chordata | Actinopterygii | Perciformes |  | Unknown fish sp1 | 1 |
| Chordata | Actinopterygii | Perciformes |  | Unknown fish sp2 | 1 |
| Chordata | Actinopterygii | Perciformes | Bathydraconidae | *Parachaenichthys charcoti* | 1 |
| Chordata | Actinopterygii | Perciformes | Channichthyidae | Channichthyidae | 5 |
| Chordata | Actinopterygii | Perciformes | Channichthyidae | Channichthyidae sp1 | 7 |
| Chordata | Actinopterygii | Perciformes | Channichthyidae | Channichthyidae sp2 | 2 |
| Chordata | Actinopterygii | Perciformes | Channichthyidae | Channichthyidae sp3 | 1 |
| Chordata | Actinopterygii | Perciformes | Channichthyidae | Channichthyidae sp4 | 1 |
| Chordata | Actinopterygii | Perciformes | Channichthyidae | *Chionodraco rastrospinosus* | 1 |

S2 Table. Continued.

| Phylum | Class | | Order | | Family | | Genus species | | Freq | |
| --- | --- | --- | --- | --- | --- | --- | --- | --- | --- | --- |
| Chordata | | Actinopterygii | | Perciformes | | Channichthyidae | | *Pagetopsis macropterus* | | 2 |
| Chordata | | Actinopterygii | | Perciformes | | Nototheniidae | | *Lepidonotothen nudifrons* | | 1 |
| Chordata | | Actinopterygii | | Perciformes | | Nototheniidae | | *Lepidonotothen* sp. | | 1 |
| Chordata | | Actinopterygii | | Perciformes | | Nototheniidae | | *Lepidonotothen squamifrons* | | 2 |
| Chordata | | Actinopterygii | | Perciformes | | Nototheniidae | | *Lindbergichthys nudifrons* | | 2 |
| Chordata | | Actinopterygii | | Perciformes | | Nototheniidae | | Nototheniidae | | 8 |
| Chordata | | Actinopterygii | | Perciformes | | Nototheniidae | | Nototheniidae sp1 | | 1 |
| Chordata | | Actinopterygii | | Perciformes | | Nototheniidae | | Nototheniidae sp2 | | 2 |
| Chordata | | Actinopterygii | | Perciformes | | Nototheniidae | | *Pleuragramma antarctica* | | 6 |
| Chordata | | Actinopterygii | | Perciformes | | Nototheniidae | | *Trematomus bernacchii* | | 1 |
| Chordata | | Actinopterygii | | Perciformes | | Nototheniidae | | *Trematomus* sp. | | 1 |
| Chordata | | Actinopterygii | | Perciformes | | Zoarcidae | | Zoarcidae sp1 | | 1 |
| Chordata | | Actinopterygii | | Perciformes | | Zoarcidae | | Zoarcidae sp2 | | 2 |
| Chordata | | Actinopterygii | | Scorpaeniformes | | Liparidae | | *Paraliparis* sp. | | 1 |
| Chordata | | Ascidiacea | | Stolidobranchia | | Pyuridae | | *Pyura bouvetensis* | | 8 |
| Chordata | | Ascidiacea | | Stolidobranchia | | Pyuridae | | *Pyura setosa* | | 1 |
| Chordata | | Aves | | Sphenisciformes | | Spheniscidae | | *Pygoscelis papua* | | 1 |
| Chordata | | Elasmobranchii | | Rajiformes | | Arhynchobatidae | | *Bathyraja maccaini* | | 1 |
| Chordata | | Mammalia | | Carnivora | | Phocidae | | *Hydrurga leptonyx* | | 3 |
| Cnidaria | | Anthozoa | | Actiniaria | |  | | Actiniaria sp1 | | 1 |
| Cnidaria | | Anthozoa | | Actiniaria | |  | | Actiniaria sp2 | | 1 |
| Cnidaria | | Anthozoa | | Actiniaria | | Hormathiidae | | Hormathia sp1 | | 1 |
| Cnidaria | | Anthozoa | | Alcyonacea | |  | | Alcyonacea sp1 | | 1 |
| Cnidaria | | Anthozoa | | Alcyonacea | |  | | Alcyonacea sp2 | | 1 |
| Cnidaria | | Anthozoa | | Alcyonacea | | Isididae | | Echinisis sp. | | 5 |
| Cnidaria | | Anthozoa | | Alcyonacea | | Primnoidae | | Primnoidae sp1 | | 1 |
| Cnidaria | | Anthozoa | | Alcyonacea | | Primnoidae | | Primnoidae sp2 | | 1 |
| Cnidaria | | Anthozoa | | Pennatulacea | |  | | Pennatulacea sp1 | | 1 |
| Cnidaria | | Hydrozoa | |  | |  | | Hydrozoa sp1 | | 1 |

S2 Table. Continued.

| Phylum | Class | | Order | | Family | | Genus species | | Freq | |
| --- | --- | --- | --- | --- | --- | --- | --- | --- | --- | --- |
| Cnidaria | | Hydrozoa | |  | |  | | Hydrozoa sp2 | | 1 |
| Cnidaria | | Hydrozoa | |  | |  | | Hydrozoa sp3 | | 1 |
| Cnidaria | | Hydrozoa | | Anthoathecata | |  | | Anthoathecata sp1 | | 2 |
| Cnidaria | | Hydrozoa | | Anthoathecata | |  | | Anthoathecata sp2 | | 1 |
| Cnidaria | | Hydrozoa | | Narcomedusae | | Solmundaeginidae | | *Solmundella bitentaculata* | | 4 |
| Cnidaria | | Hydrozoa | | Siphonophorae | |  | | Siphonophorae sp1 | | 2 |
| Cnidaria | | Hydrozoa | | Siphonophorae | |  | | Siphonophorae sp2 | | 1 |
| Cnidaria | | Hydrozoa | | Trachymedusae | |  | | Trachymedusae sp1 | | 1 |
| Cnidaria | | Hydrozoa | | Trachymedusae | |  | | Trachymedusae sp2 | | 1 |
| Cnidaria | | Hydrozoa | | Trachymedusae | | Ptychogastriidae | | *Ptychogastria polaris* | | 3 |
| Cnidaria | | Scyphozoa | |  | |  | | Scyphozoa sp1 | | 1 |
| Ctenophora | | Nuda | | Beroida | | Beroidae | | *Beroe cucumis* | | 1 |
| Ctenophora | | Tentaculata | | Platyctenida | | Lyroctenidae | | *Lyrocteis flavopallidus* | | 2 |
| Echinodermata | | Asteroidea | |  | |  | | Asteroidea sp1 | | 1 |
| Echinodermata | | Asteroidea | |  | |  | | Asteroidea sp2 | | 2 |
| Echinodermata | | Asteroidea | | Forcipulatida | |  | | Forcipulatida sp1 | | 3 |
| Echinodermata | | Asteroidea | | Forcipulatida | | Asteriidae | | *Diplasterias brucei* | | 3 |
| Echinodermata | | Asteroidea | | Forcipulatida | | Asteriidae | | *Diplasterias* sp. | | 1 |
| Echinodermata | | Asteroidea | | Spinulosida | | Echinasteridae | | *Henricia* sp1 | | 1 |
| Echinodermata | | Asteroidea | | Valvatida | | Ganeriidae | | *Perknaster* sp. | | 1 |
| Echinodermata | | Asteroidea | | Valvatida | | Odontasteridae | | *Acodontaster* sp. | | 1 |
| Echinodermata | | Asteroidea | | Valvatida | | Poraniidae | | *Glabraster antarctica* | | 4 |
| Echinodermata | | Crinoidea | |  | |  | | Crinoidea sp1 | | 1 |
| Echinodermata | | Crinoidea | |  | |  | | Crinoidea sp2 | | 1 |
| Echinodermata | | Crinoidea | | Comatulida | |  | | Comatulid sp1 | | 1 |
| Echinodermata | | Crinoidea | | Comatulida | | Antedonidae | | *Solanometra antarctica* | | 1 |
| Echinodermata | | Crinoidea | | Comatulida | | Antedonidae | | *Solanometra* sp1 | | 1 |
| Echinodermata | | Echinoidea | | Camarodonta | | Echinidae | | *Sterechinus neumayeri* | | 2 |

S2 Table. Continued.

| Phylum | Class | | Order | | Family | | Genus species | | Freq | |
| --- | --- | --- | --- | --- | --- | --- | --- | --- | --- | --- |
| Echinodermata | | Echinoidea | | Cidaroida | |  | | Cidaroida sp1 | | 1 |
| Echinodermata | | Echinoidea | | Cidaroida | |  | | Cidaroida sp2 | | 1 |
| Echinodermata | | Echinoidea | | Cidaroida | | Ctenocidaridae | | *Ctenocidaris perrieri* | | 7 |
| Echinodermata | | Holothuroidea | | Elasipodida | | Elpidiidae | | *Peniagone* sp. | | 5 |
| Echinodermata | | Ophiuroidea | | Ophiurida | | Ophiopyrgidae | | *Ophioperla koehleri* | | 2 |
| Echinodermata | | Ophiuroidea | | Ophiurida | | Ophiuridae | | *Ophionotus victoriae* | | 11 |
| Echinodermata | | Ophiuroidea | | Ophiurida | | Ophiuridae | | *Ophiosparte gigas* | | 2 |
| Mollusca | | Cephalopoda | | Octopoda | | Megaleledonidae | | Pareledone sp. | | 2 |
| Mollusca | | Cephalopoda | | Oegopsida | |  | | Oegopsida sp1 | | 7 |
| Mollusca | | Cephalopoda | | Oegopsida | | Psychroteuthidae | | *Psychroteuthis glacialis* | | 8 |
| Mollusca | | Gastropoda | | Neogastropoda | | Volutidae | | Volutidae sp1 | | 3 |
| Nemertea | |  | |  | |  | | Nemertea sp1 | | 1 |
| Nemertea | | Pilidiophora | | Heteronemertea | |  | | Heteronemertea sp1 | | 2 |
| Nemertea | | Pilidiophora | | Heteronemertea | | Lineidae | | *Parborlasia corrugatus* | | 1 |
| Porifera | | Demospongiae | |  | |  | | Demospongiae sp1 | | 1 |
| Porifera | | Demospongiae | | Haplosclerida | | Chalinidae | | *Haliclona* sp1 | | 1 |
| Porifera | | Demospongiae | | Poecilosclerida | | Mycalidae | | *Mycale* (Oxymycale) acerata | | 1 |
| Porifera | | Demospongiae | | Suberitida | | Suberitidae | | *Suberites* sp1 | | 1 |
| Porifera | | Demospongiae | | Tetractinellida | | Tetillidae | | *Cinachyra antarctica* | | 1 |
| Porifera | | Hexactinellida | |  | |  | | Hexactinellida sp1 | | 1 |
| Porifera | | Hexactinellida | | Lyssacinosida | | Rossellidae | | *Rossella nuda* | | 1 |
| Porifera | | Hexactinellida | | Lyssacinosida | | Rossellidae | | *Rossella podagrosa* | | 1 |
| Porifera | | Hexactinellida | | Lyssacinosida | | Rossellidae | | *Rossella* sp1 | | 1 |
| Porifera | | Hexactinellida | | Lyssacinosida | | Rossellidae | | *Rossella* sp2 | | 1 |
| Porifera | | Hexactinellida | | Lyssacinosida | | Rossellidae | | Rossellidae sp1 | | 1 |
